# Generation of megahertz-band spin currents using nonlinear spin pumping

**DOI:** 10.1038/s41598-017-04901-4

**Published:** 2017-07-04

**Authors:** Shingo Watanabe, Daichi Hirobe, Yuki Shiomi, Ryo Iguchi, Shunsuke Daimon, Mai Kameda, Saburo Takahashi, Eiji Saitoh

**Affiliations:** 1 Institute for Materials Research, Tohoku University, Sendai, 980-8577 Japan; 2 WPI Advanced Institute for Materials Research, Tohoku University, Sendai, 980-8577 Japan; 3 Advanced Science Research Center, Japan Atomic Energy Agency, Tokai, 319-1195 Japan

## Abstract

Spin pumping enables the generation of d.c. and gigahertz-band (GHz-band) voltages from an applied microwave via magnetization dynamics when combined with inverse spin Hall effects. However, generating such voltages in the in-between frequency region, or the megahertz (MHz) band, has been difficult since ferromagnetic resonance usually occurs in the GHz band. Here we show that in spite of GHz-band microwaves applied, MHz-band voltages can be generated by spin pumping with use of nonlinear magnetization dynamics in Y_3_Fe_5_O_12_. The mechanism is ascribed to the MHz-band oscillation of the amplitude of the magnetization precession, which is projected onto a rectified voltage component via spin pumping. The present finding could be useful for frequency down-conversion thanks to the simple and durable structure, continuous-wave operation, and the tunability of an output frequency with low magnetic fields.

## Introduction

Spin current is a flow of spin angular momentum of electrons in solids without accompanying charge current^1^, which has attracted much attention in spintronics^2, 3^. Since spin current can be generated also in magnetic insulators, insulator-based spintronic phenomena, such as spin-torque and spin Seebeck effects, have been explored^4–9^.

**Figure 1:**
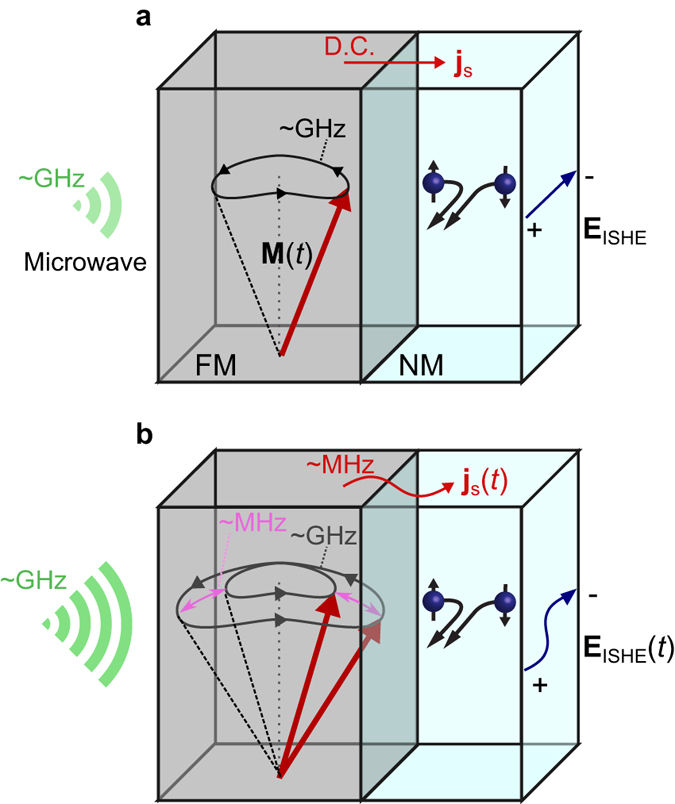
Concept of generation of MHz-band spin currents by nonlinear spin pumping. (**a,b**) Spin pumping at the interface between ferromagnetic material (FM) and nonmagnetic metal (NM) in a linear regime (**a**) and an auto-oscillation (AO) regime (**b**) of magnetization dynamics. When the power of an incident microwave is below the threshold value of AO (**a**), the precessing magnetization, **M**(*t*), injects a d.c. spin current, **j**_s_, into the NM via the spin pumping effect, which results in the d.c. electromotive force **E**_ISHE_ via the inverse spin Hall effect (ISHE). When the microwave power exceeds the threshold value of AO (**b**), the solid angle subtended by **M**(*t*) exhibits AO. As a result, a MHz-band spin current, **j**_s_(*t*), arises from spin pumping, which is finally converted into pulsating electromotive force, **E**_ISHE_(*t*) via ISHE.

Among various spin-current generation methods, spin pumping is a versatile way to generate spin current in a ferromagnetic material (FM)/nonmagnetic metal (NM) structure^10, 11^. When steady magnetization precession dynamics is excited in a FM, a d.c. spin current is injected from the FM into the NM through the interface, a phenomenon called spin pumping (Fig. 1a). The magnitude of spin current is proportional to the square of the magnetization-precession amplitude^1^. By using a NM with strong spin-orbit interaction, such as platinum (Pt), the spin current injected in the NM is converted into voltage via the inverse spin Hall effect^12, 13^ (ISHE) caused by the spin-orbit interaction^14–21^.

Magnetization in a FM, such as a magnetic insulator Y_3_Fe_5_O_12_ (YIG), is known to exhibit typical nonlinear dynamics; when a strong microwave is applied to a FM, a steady magnetization precession motion becomes unstable^22^, and auto-oscillation (AO) of the precession amplitude shows up as a nonlinear turbulent bifurcation effect when the microwave power is greater than the AO threshold power^23–31^. By further increasing the microwave power, the magnetization dynamics then collapses into a chaos phase^32^. Here, the AO frequency Ω is much less than the magnetization-precession frequency, in general^23–31^. However, the AO is an oscillation of the amplitude in the precession motion, and it does not contain a wave component of the frequency Ω; the AO was difficult to utilize.

Here we show that the AO of the precession amplitude can be converted into a voltage oscillation of a much lower frequency, Ω, inside a material exhibiting spin pumping (Fig. 1b). Spin current induced by spin pumping reflects the amplitude of the magnetization precession^1^, and spin pumping thus converts an amplitude oscillation into an oscillation of spin current, which can be converted into a voltage oscillation by using ISHE. By introducing spin pumping and ISHE into magnetization AO, we demonstrated huge frequency conversion from a microwave with the magnetization-precession frequency into an electric voltage oscillation with the frequency Ω.

## Results

### Ferromagnetic resonance in Pt/YIG

**Figure 2:**
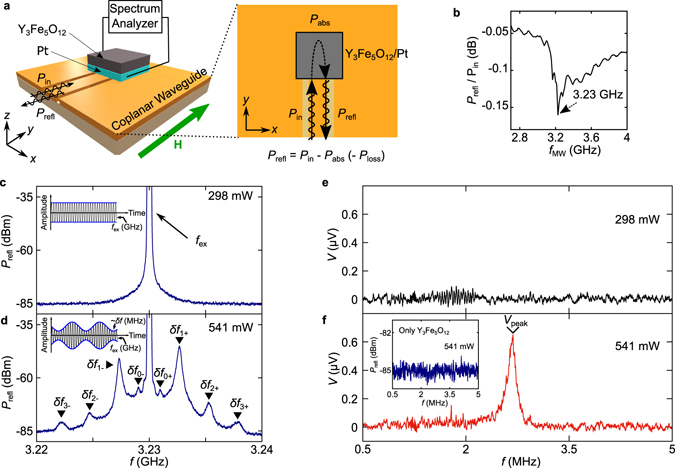
Generation of MHz-band voltages from GHz-band microwaves. (**a**) Schematic illustration of the experimental set-up for the ferromagnetic resonance (FMR) and spin pumping measurements. **H** denotes the static magnetic field; *P*_in_ the power of the incident microwave; *P*_refl_ the reflected microwave; *P*_abs_ the absorbed microwave by spin-wave resonance in Y_3_Fe_5_O_12_; *P*_loss_ the return loss on the equipment. (**b**) Microwave frequency, *f*_MW_, dependence of the microwave absorption *P*_refl_ */P*_in_. The *P*_in_ and the magnitude of **H** (*H*) were set at 541 mW and 545 Oe, respectively. (**c**,**d**) Microwave-power dependence of the reflected microwave at *P*_in_ = 298 mW (**c**) and 541 mW (**d**). The most prominent excitation frequency (*f*_ex_) is 3.23 GHz. *H* was set at 545 Oe. In 541 mW, δ*f*_0±_, δ*f*_1±_, δ*f*_2±_ and δ*f*_3±_ are the sidepeak frequencies of reflected microwaves by the AO. The δ*f*_1_ is the fundamental frequency of the AO. δ*f*_2_ and δ*f*_3_ are higher harmonic components of δ*f*_1_. δ*f*_0_ is a subharmonic component of δ*f*_1_. The insets to (**c**) and (**d**) show schematic illustrations of the waveforms of the output microwave at *P*_in_ = 298 mW (**c**, AO is off) and 541 mW (**d**, AO is on). *P*_refl_ becomes the amplitude-modulated wave by the AO. (**e,f**) Voltage, *V*, spectra for the Pt/Y_3_Fe_5_O_12_ film at *P*_in_ = 298 mW (**e**) and 541 mW (**f**). The frequency of the voltage is approximately 2.7 MHz. The inset to (**f**) shows the frequency spectrum of *P*_refl_ at *P*_in_ = 541 mW.

Figure 2a is a schematic illustration of the experimental set-up used in the present study. The sample consists of a single crystalline film of ferromagnetic insulator YIG (FM) on the top of which a polycrystalline Pt thin film (NM) is put**;** the Pt is used as a spin-current detector based on the ISHE. A microwave with the power *P*_in_ was irradiated onto the sample with applying a static in-plane magnetic field (*H*). Power spectra of the reflected microwave from the sample, *P*_refl_, and the frequency spectrum of the voltage *V* generated between the ends of the Pt film were measured using a spectrum analyzer. We confirmed conventional d.c. ISHE voltages due to spin pumping by replacing the spectrum analyzer with a nanovoltmeter (see Supplementary Fig. S1).

Figure 2b shows a microwave reflection spectrum, *P*_refl_/*P*_in_, measured for the Pt/YIG film at *P*_in_ = 541 mW while sweeping the input microwave frequency *f*_MW_. A dip structure is clearly observed at 3.23 GHz. The dip shows that the absorption of the applied microwave is maximized at 3.23 GHz corresponding to the ferromagnetic resonance (FMR) excitation in the YIG. Hereafter, the values of the input microwave frequency and the external magnetic field were set to those in this FMR condition (*f*_ex_ = 3.23 GHz and *H* = 545 Oe).

### Auto-oscillation of magnetization

Figure 2c and d show the frequency power spectrum of the microwave *P*_refl_ transmitted back from the sample, measured in the FMR condition (*f*_ex_ = 3.23 GHz). At *P*_in_ = 298 mW, a single peak appears at 3.23 GHz, the same frequency as the incident microwave *f*_ex_, which is a microwave reflected directly by the sample (see Fig. 2c). On the other hand, the spectra of *P*_refl_ show a dramatic change when the incident microwave power is increased to induce nonlinear magnetization dynamics. With increasing *P*_in_ to 541 mW, sidepeaks, labeled by δ*f*, arose around the main peak at *f*_ex_ = 3.23 GHz in the *P*_refl_ spectrum (see Fig. 2d). The values of the frequency displacement of the sidepeaks from the main peak are about 0.9 MHz (δ*f*_0±_), 2.7 MHz (δ*f*_1±_), 5.4 MHz (δ*f*_2±_), and 8.1 MHz (δ*f*_3±_), which exhibit a clear periodicity. The appearance of the sidepeaks in the spectrum shows that auto-oscillation^23–30^ (AO) with the frequency δ*f* appears at *P*_in_ = 541 mW. At FMR along with AO, the precession trajectory of the YIG magnetization is periodically modulated with the MHz frequencies, which in turn modulates the FMR absorption intensity with the same MHz frequency. Hence, the amplitude of the output microwave is modulated with the MHz frequency, as shown in the inset of Fig. 2d.

### MHz voltage due to spin pumping by auto-oscillation of magnetization

In the FMR condition, we measured frequency spectra of *V* between the ends of the Pt layer to detect spin-pumping ISHE voltage. Figure 2e and f show the *V* spectra measured in the MHz range. At *P*_in_ = 298 mW (Fig. 2e), no voltage signal appears in *V* in the entire MHz range, consistent with the previous reports on ISHE induced by spin pumping^14–21^. On the other hand, importantly, with increasing *P*_in_ to 541 mW (Fig. 2f), a voltage peak *V*_peak_ shows up at Ω = 2.7 MHz in spite of the application of a GHz microwave. The peak amplitude *V*_peak_ is about 0.66 μV, comparable to the typical d.c. ISHE voltage in literatures^14–21^. Although the AO itself does not produce MHz wave components as shown in the inset to Fig. 2f, the Pt/YIG can convert the AO into the MHz electric voltage. We note that the reproducibility of the MHz-band voltage was confirmed by replacing the sample and performing the same measurement several times (see Supplementary Fig. S2). It is also noted that the ISHE voltage was not observed at *f*_ex_ + δ*f*_2±_ or *f*_ex_ + δ*f*_3±_; these voltage signals seem too small to be observed within our experimental accuracy (see also Fig. 2d for much smaller absorption peaks at those higher harmonics in the reflected microwave spectrum).

### Absence of MHz voltage in Pt/SiO_2_/YIG

**Figure 3:**
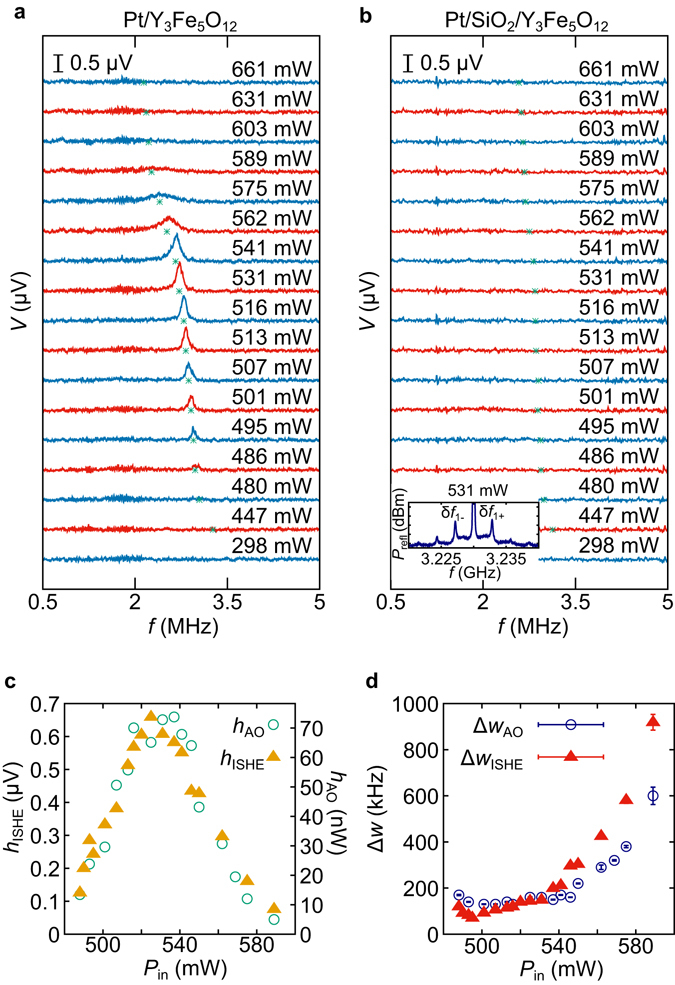
AO-induced spin currents in MHz-band. (**a**,**b**) Frequency dependence of *V* in the MHz range in Pt/Y_3_Fe_5_O_12_ (**a**) and Pt/SiO_2_/Y_3_Fe_5_O_12_ (**b**) at various values of *P*_in_. Green points indicate frequencies of the AO, δ*f*_1+_, at each *P*_in_ (see Fig. 2d). The inset to (**b**) shows the *P*_refl_ spectrum for the Pt/SiO_2_/Y_3_Fe_5_O_12_ film at *P*_in_ = 531 mW. The incident microwave frequency was 3.23 GHz. *H* was set at 545 Oe. (**c**) *P*_in_ dependence of the peak height *h*_AO_ of the GHz AO sidepeak at δ*f*_1+_ (*h*_AO_) and the MHz-band voltage peak (*h*_ISHE_), measured in the Pt/Y_3_Fe_5_O_12_ film (**a**). (**d**) *P*_in_ dependence of the half width of the peak at δ*f*_1+_ for the GHz AO sidepeak, Δ*w*_AO_, (Fig. 2d) and the MHz-band voltage peak, Δ*w*_ISHE_, (**a**).

To further confirm that the voltage peak at 2.7 MHz (Fig. 2f) originates from the spin pumping effect, a similar experiment was performed for Pt/SiO_2_/YIG, in which the SiO_2_ film prevents spin-current injection from YIG into Pt. Figure 3a and b compare the MHz-band voltage spectra between Pt/YIG and Pt/SiO_2_/YIG at various *P*_in_ values. For Pt/SiO_2_/YIG, the voltage peak was found to disappear in the whole range of *P*_in_, while it is clearly observed for Pt/YIG in *P*_in_ = 486–589 mW. Since the AO still appears in the *P*_refl_ spectra for Pt/SiO_2_/YIG (see inset to Fig. 3b), the disappearance of the voltage peak shows that the spin exchange at the Pt/YIG interface is essential for the MHz voltage peak to appear. Hence, the voltage peak observed in Pt/YIG is attributed to MHz-band spin current due to spin pumping, not to other trivial origin, e.g. electromagnetic induction. We note that a rectification effect of magnetization dynamics based on spin-Hall magnetoresistance^33, 34^ may give rise to MHz voltage in the present experimental set-up; however, such a component is expected to be much smaller than that of spin pumping on the basis of experimental and theoretical studies on d.c. voltages rectified^35–37^. Separating these two effects quantitatively under auto-oscillation conditions is open to future studies.

### Dependence on power levels of incident microwave

In Fig. 3c and d, the peak shapes are compared between the main AO microwave signal at δ*f*_1+_ (Fig. 2d) and the MHz-band voltage peak (Fig. 3a) at various *P*_in_ values, to further corroborate that the spin pumping drives the GHz-MHz down-conversion. The peak height *h* and the half width at the half maximum Δ*w* are obtained by fitting the peaks to Lorentzian functions, and are plotted as functions of the incident microwave power *P*_in_ in Fig. 3c and d, respectively. As shown in Fig. 3c, the peak height *h* shows the same *P*_in_ dependence for the AO microwave signal and the voltage signal in the entire *P*_in_ range: they reach their maximums at about 525 mW, and then decrease towards zero with further increasing *P*_in_. Also, Fig. 3d shows that the half width Δ*w* for the main AO microwave signal at δ*f*_1+_ coincides with that for the voltage peak in the whole *P*_in_ range. The similar *P*_in_ dependence implies that the AO gives rise to the MHz-band voltage via the spin pumping effect. It is noted that Δ*w* increases monotonically with *P*_in_. This broadening can be attributed to the gradual transition from the periodic AO to chaotic phase^23–27, 31^ at the very high *P*_in_ values.

## Discussion

In summary, we have demonstrated the generation of MHz-band spin currents using spin pumping from nonlinear magnetization dynamics of YIG. The generated MHz-band spin currents were converted into electric voltages using ISHE in Pt, which can enable frequency down-conversion from a GHz-band input microwave into a MHz-band output signal. The observed frequency down-conversion due to a spin current may have several advantages for future device application, though the conversion efficiency needs to be improved. First, Pt/YIG can down-convert a wide-range of microwaves since the mechanism is based on FMR. Second, the output frequency may be also tunable in a wide frequency range, from kHz to MHz, by changing the static magnetic field and the magnetization damping in the FM^23–28^. Such tunability within a MHz region was demonstrated as seen in Supplementary Fig. S3 although a window of the frequency tunability was narrow in the present study (~0.8 MHz). Third, the material system is a simple thin-film bilayer structure without using complex heterodyne circuits^38^. Nonlinear properties of the spin-current generation may realize a new scheme of material design for next-generation spintronics devices.

## Methods

### Sample preparation

A single crystalline Y_3_Fe_5_O_12_ (111) film (19-µm thick) was grown on a Gd_3_Ga_5_O_12_ (111) substrate (500-µm thick) by liquid phase epitaxy. The Y_3_Fe_5_O_12_ */*Gd_3_Ga_5_O_12_ sample was cut into rectangles with the surface area of 1 × 1 mm^2^. The surface of the Y_3_Fe_5_O_12_ film was polished mechanically before the sputtering of Pt and SiO_2_ films. The Pt film (6-nm thick) was sputtered in an Ar atmosphere; the SiO_2_ film (20-nm thick) was sputtered in an Ar and O_2_ atmosphere in ratio of Ar:O_2_ = 10:3 in volume.

### Spin pumping measurement

A coplanar waveguide was used to apply a microwave to the Pt/Y_3_Fe_5_O_12_ sample. The signal line of the coplanar waveguide was 200 µm wide, designed to match the characteristic impedance of 50 Ω. The microwave magnetic fields (3.23 GHz) and static magnetic fields (545 Oe) were applied perpendicular to each other within the plane of Y_3_Fe_5_O_12_ film. The microwave absorption *P*_refl_/*P*_in_ was measured with a vector network analyzer (Agilent Technologies N5230N); the microwave transmittance with a spectrum analyzer (Agilent Technologies N9010A). When the incident microwave frequency is swept, d.c. voltage measured using a nanovoltmeter (Keithley 2182 A) shows a peak at *f*_ex_ = 3.23 GHz, and exhibits sign reversal by reversing the magnetic field direction (see also Supplementary Information). The MHz-band voltage was amplified using a low noise preamplifier and measured using a spectrum analyzer. All of the measurements were performed at room temperature.

## Electronic supplementary material

Supplementary Information
