# Supplementary material for: Generation of megahertz-band spin currents using nonlinear spin pumping
Source: Sci Rep. 2017 Jul 4;7:4576. doi: 10.1038/s41598-017-04901-4 (PMC5496877; doi:10.1038/s41598-017-04901-4)
Supplement: Supplementary file 1 — Supplementary Information [file 41598_2017_4901_MOESM1_ESM.pdf]

Supplementary Information for Generation of megahertz-band spin currents using nonlinear spin pumping

Shingo Watanabe[1], Daichi Hirobe[1], Yuki Shiomi[1], Ryo Iguchi[1], Shunsuke Daimon[1,2], Mai Kameda[1], Saburo Takahashi[1], and Eiji Saitoh[1,2,3]

[1]*Institute for Materials Research, Tohoku University, Sendai 980-8577, Japan*

[2]*WPI Advanced Institute for Materials Research, Tohoku University, Sendai 980-8577, Japan*

[3]Advanced Science Research Center, Japan Atomic Energy Agency, Tokai 319-1195, Japan

(i) d.c. ISHE in Pt/$Y_3Fe_5O_{12}$

In Fig. S1, we show the d.c. voltage $V^{\mathrm{d.c.}}$ measured between the ends of the Pt film as a function of the incident microwave frequency. The measurement was performed in the set-up shown in Fig. 2a with the spectrum analyzer replaced with a nanovoltmeter. Clear voltage peaks are observed at f_{MW} = 3.23 GHz, which corresponds to the FMR frequency of the $Y_3Fe_5O_{12}$. Since the sign of $V^{\mathrm{d.c.}}$ peak is reversed by reversing the polarity of the magnetic field, the observed $V^{\mathrm{d.c.}}$ peaks are attributed to spin current injected from the $Y_3Fe_5O_{12}$ via spin pumping[14].

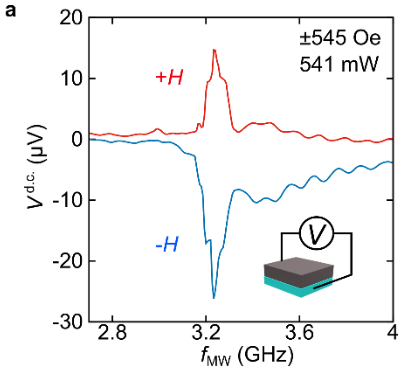

Fig. S1: **d.c. ISHE voltage in Pt/$Y_3Fe_5O_{12}$.**

a, Microwave frequency, f_{MW}, dependence of the d.c. voltage $V^{\mathrm{d.c.}}$ for the Pt/$Y_3Fe_5O_{12}$ film at the incident power P_{in} = 541mW.

(ii) Reproducibility of MHz-band voltages

We checked the reproducibility of our experimental results by remounting the sample several times. A representative result is shown in Fig. S2, in which the spin pumping signal was found to be reproducible. However, there was a change in the threshold power P_{th} for the auto-oscillation of the magnetization: P_{th} was ~350 mW (~480 mW) for the measurement presented in Fig. S2 (3a). This change might be attributed to an inevitable change in sample alignment.

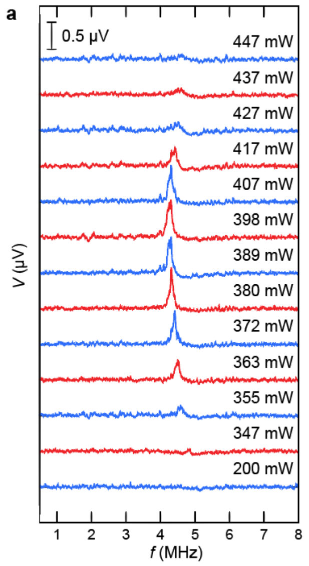

Fig. S2: **Reproducibility of MHz-band voltages caused by spin pumping.**

a, Frequency (f) spectra of the MHz-band voltage (V) measured in Pt/ $Y_3Fe_5O_{12}$ at various values of the incident power. Values of the incident microwave frequency and the static field were the same as those set for the result in Fig. 3a.

(iii) Tunability of frequency of MHz-band voltage by external magnetic field

In Fig. S3, we show the field dependence of the output frequency of MHz-band voltage, investigated in the set-up shown in Fig. 2a in the main text. Increasing the field from 543 Oe to 547 Oe was found to increase the output frequency from ~2.35 MHz to ~3.17 MHz. The result points to the tunability of the MHz-band voltage by external magnetic fields. We note, however, that a window of the frequency tunability was narrow (~0.8 MHz).

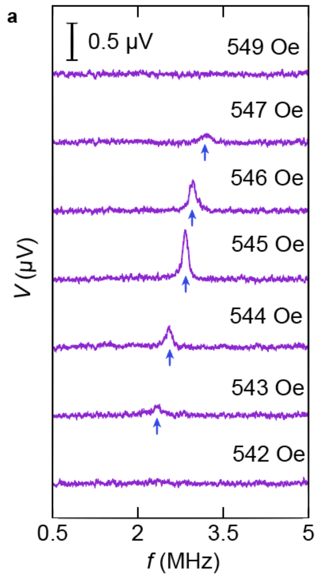

Fig. S3: **Tunability of output frequency of MHz-band ISHE voltage by magnetic field.**

a, Frequency (f) spectra of the MHz-band voltage (V) measured in Pt/ $Y_3Fe_5O_{12}$ for several magnetic fields. The incident power was set at 541 mW.
